# The smooth and bumpy road of trastuzumab administration: from intravenous (IV) in a hospital to subcutaneous (SC) at home

**Published:** 2017-03

**Authors:** WAA Tjalma, MT Huizing, K Papadimitriou

**Affiliations:** Multidisciplinary Breast Clinic, Gynaecological Oncology Unit, Department of Obstetrics and Gynaecology, Antwerp; University Hospital - University of Antwerp, 2650 Antwerpen, Belgium

**Keywords:** Breast cancer, trastuzumab, intravenous, subcutaneous, home, hospital

## Abstract

Trastuzumab has become standard of care in the treatment of early and metastatic HER2-positive breast cancer. Initially trastuzumab could only be administered intravenously (IV), however since a few years there is also a subcutaneous (SC) formulation. The efficacy and the safety profile of both formulations is the comparable. The administration logistics however have an impact on the patients, the health care professionals (HCPs), the hospital and the government. The preference for the patients (89%) and the HCPs (77%) is in favour of the SC formulation. The patient chair time per cycle, as defined by the time between entry and exit of infusion chair, is between 53 and 122 minutes shorter for SC administration. Also, the time actively dedicated by the HCP on preparation and administration SC, is between 17 and 50 minutes shorter per cycle. These time savings may increase the capacity of an oncological day clinic and reduce waiting lists. An additional benefit is that the use of SC formulation reduces the consumables and the waste. When the SC form was given at home instead of in the hospital the safety profile remained the same, but the satisfaction rate improved further for both the patients and the HCPs. The next and final step will be potentially to invest in teaching the patients to self-administer the medication. The home administration and the education of the patients and the HCPs will have a cost price and it will be interesting to see how the hospital financial authorities and the government will deal with this situation in the time of budgetary restrictions.

## Introduction

Trastuzumab (Herceptin R) is part of the standard of care in breast cancer patients with a HER2 receptor overexpression. Both in early and in metastatic setting treatment with trastuzumab increased survival. In early dates trastuzumab was administrated intravenously (IV), but since 3 years it can also be given subcutaneously (SC) (Wynne et al., 2013; Papadimitriou et al., 2015). The route of administration has no influence on the pharmacokinetics and the efficacy is comparable for both routes (Papadmitriou et al., 2015). Trastuzumab usage is also related to side effects. The most serious and commonly reported adverse events include cardiac dysfunction, administration related reactions, haematotoxicity (in particular neutropenia), infections and pulmonary adverse reactions (EMA, 2017). There is no significant difference concerning the severe adverse events (defined according to the National Cancer Institute Common Terminology Criteria for Adverse Events (NCI CTCAE grade ≥3) version 3.0)) between the two formulations. Adverse events however, are seen more frequently in the SC formulation: infections with or without neutropenia (4.4 % IV versus 8.1 % SC); cardiac disorders (0.7 % IV versus 1.7 % SC); post-operative wound infections (1.7 % IV versus 3.0 % SC); administration-related reactions (37.2 % IV versus 47.8 % SC); hypertension (4.7 % IV versus 9.8 % SC) (EMA, 2017). In Table I the differences between the two formulations are summarized. In the product characteristics of EMA it is clearly stated how trastuzumab should be administrated (EMA, 2017); for IV trastuzumab the first administration (loading dose) is over 90 minutes and the patients should be observed for at least six hours after the start of infusion. The administration should be done under the supervision of a healthcare provider prepared to manage anaphylaxis and an emergency kit should be available (EMA, 2017). Severe anaphylactic reactions occur usually during either the first or second infusion of trastuzumab and have been occasionally associated with a fatal outcome (EMA, 2017). The subsequent doses should be given as a 30-minute infusion and the patient should be observed for two hours. The SC administration should be done in the thigh over a 2 - 5 minutes period and the patients should be observed for six hours after the first injection and for two hours after the subsequent injections (EMA, 2017).

**Table I:** Comparing Herceptin form IV and SC

		IV	SC
Schedule		3-weekly	3-weekly
Vial		150 mg	600 mg
Price of 1 vial		556,02 €	1684,30 €
Weight-based dose calculation		Yes	No
Loading dose		Yes	No
	- Initial dose	8 mg/kg	NA
	- Initial infusion time	90 min	NA
	- Maintenance dose	6 mg/kg	NA
	- Maintenance infusion time*	30 min	NA
Injection time Observation time after administration		NA	2-5 min
	- 1ste dose	6 hours	6 hours
	- Subsequent after preparing	2 hours	2 hours
	- Stable 48 hours at 2°C-8°C	Yes	Yes
	- Stable 6 hours in daylight (&30°C)	NA	Yes
	Remaining solution should be discarded	Yes	NA

*If the initial dose was well tolerated than the administration time can be reduced; NA = not applicable

The Food and Drug administration approved trastuzumab as the first targeted therapy for HER2- positive breast cancer, but with a high price tag. Indeed, the ex-factory price (exclusive BTW) of trastuzumab in Belgium is €517,83 and €1582,25 for the IV and SC formulation respectively (RIZIV, 2017). The reimbursement in Belgium includes extra costs for an ambulant patient and 6 % BTW for the IV formulation resulting in a cost of €278,01 (per half vial of 75 mg), while for the SC formulation it is €1684,30. In the adjuvant setting (18 cycles), the cost comparison between SC and IV formulation favours the SC formulation for women with a weight > 75 kg, while for patients with a weight below 75 kg IV formulation is cheaper (Table II).

**Table II:** Price calculation of adjuvant trastuzumab (18 cycles) per weight categories

Weight (kilogram)	87,5	84	75	62,5	56,25	50
IV loading dose vials	5	4,5	4	3,5	3	3
IV subsequent dose	3,5	3,5	3	2,5	2,5	2
Total vials	64,5	64	55	46	45,5	37
Price IV (€)	33400,04	33141,12	28480,65	23820,18	23561,27	19157,71
Price SC (€)	28480,50	28480,50	28480,50	28480,50	28480,50	28480,50
**Difference IV – SC (€)**	**+ 4919,54**	**+ 4660,62**	**+ 0,15**	**- 4660,32**	**- 4919,24**	**- 9320,79**

½ vial = >0,0 and < 0,5 or >0,5 and < 1,0; Loading dose IV in number of vials (weight (kg) x 8mg)/150; Subsequent dose IV in number of vials (weight (kg) x 6 mg)/150; Total vials (18 cycles) = Loading number of vials + 17 x Subsequent number of vials; 1 IV vial is 517,83€; 1 SC vial is 1582,25€.

Trastuzumab is on the WHO Model List of Essential Medicines (WHO essential medicine list, 2017). The definition of essential medicine includes drugs that satisfy the health care needs of the majority of the population. Medication on the list should therefore always be available always in adequate amounts and in appropriate dosage forms, at a price the community can afford (WHO essential medicine list, 2017). 

During the San Antonio Breast Cancer Symposium 2016 there was much debate about the workload of trastuzumab administration and the ways to reduce this. If we intend to address this issue we should look at it from the perspective of the 4 stakeholders involved (Patient, Health Care professionals, Hospital and Government). Current discussion will focus on the use of trastuzumab in the adjuvant setting for early breast cancer patients.

## The patient perspective

In the PrefHer study 89% of the patients preferred the SC administration, while 2 % had no preference (Pivot et al., 2014). The patient chair time per cycle, as defined by the time between entry and exit of infusion chair, is between 53 and 122 minutes shorter for SC formulation (Pivot et al., 2014; Tjalma et al., 2016). The next step forward, as a logical evolution of trastuzumab administration, would be the administration of trastuzumab subcutaneously at home by HCPs. The BELIS (BELgium and ISrael) is the first trial for the evaluation of trastuzumab SC administration at home.

The BELIS study looked at the safety and tolerability of at home administration of SC trastuzumab for the treatment of patients with HER2-positive early breast cancer (Cocquyt et al., 2016). In this study 2577 patients were included; the home administration of SC trastuzumab had a comparable safety profile with the IV administration. At Home SC (H SC) trastuzumab was administrated in 1867 patients with a handle hold syringe by an HCP and in 710 patients via a supervised selfadministration with a single - use injection device. The adverse event (AE) rates varied between 7 -31 %, according to the timing of the chemotherapy. The HSC was satisfactory to the patient in a very large extent (83 %) and to a large extent (17%). The treatment sessions of trastuzumab were described as acceptable in 96 % of H SC, in 92 % of SC in the hospital and 80 % when given as IV in the hospital. In total 22,2% of patients reported to experience a large benefit of drug administration at home, while 77,8% reported a very large benefit (Cocquyt et al., 2016).

## The HCPs perspective

In the PrefHer study 77 % of the HCPs preferred the SC administration and 20 % had no preference (Pivot et al., 2014). The time actively dedicated by any HCP on preparation and administration SC, is between 17 and 50 minutes shorter per cycle ( Pivot et al., 2014; Tjalma et al., 2016). The BELIS study concluded that from a HCPs point of view, administration of SC was fairly easy. The outcome assessment of HCPs in the BELIS study reported that the duration of SC was less than two hours in 67 % and between two and three hours in 33 % (Cocquyt et al., 2016).

The results of the BELIS study, evaluating the overall safety and tolerability of subcutaneous (SC) trastuzumab administered at home for the treatment of patients with HER2-positive early breast cancer, indicated that trastuzumab SC was not associated with any new safety events. Moreover, trastuzumab SC was considered beneficial by the patients and health care professionals. Healthcare professionals considered SC administration to be quicker and demanding fewer preparation resources than IV. 

## The Hospital perspective

The transition from an IV to a SC formulation of trastuzumab in a hospital would reduce the chair/ bed occupation time by 326 % to 1200 %. In other words, it would increase the capacity for trastuzumab treatment by 3 to 12 times. Practically, the waiting list for all day care treatments would be reduced. In a LEAN working oncology day-care an additional benefit could be assumed for the spared chair/bed occupation for other treatments.

## The Government perspective

With the use of the SC formulation there would be no drug waste, while with the use of the IV formulation, which is calculated by weight, there is mostly a considerable drug waste. In one vial there are 150 mg of medication. If a patient’s weight is 85 kg, then she should receive 510 mg in the maintenance therapy (6 mg/kg). This would mean that 4 vials would be used (4 X 150 = 600mg). The calculated waste would then be then 90 mg or 334 euro. In Table II the calculations are shown for different weights.

## Discussion

Giving trastuzumab SC instead of IV in a hospital setting appears to have a benefit for all stakeholders.

When the SC form was given at home instead of in the hospital the safety remained the same, but the satisfactory rate increased further for the patients and the HCPs. The next and final step will be to teach the patients to inject themselves. By altering the trastuzumab formulation from IV to SC between 50 and 120 minutes are spared for the patients. From the day care unit’s point of view the bed/chair occupation time is reduced by the same amount. The occupation time is annulated if the administration occurs at home. Financially, this is interesting for the hospital because there is no real reimbursement for trastuzumab. Additionally, the HCPs resources can now be used for other patients and activities. An advantage of giving SC trastuzumab at home is the increased bonding of the first and second line caregivers.

There are several aspects and hurdles, which should be taken in consideration. Due to the possible adverse events, it should be advised to give the first two cycles in a hospital setting. The administration at home can be done by the hospital nurses, the home care nurse or a general practitioner (GP). When giving SC at home, patients must be educated about possible adverse events and there should be a control or observation system. An observation system could be for instance based on texting on well-defined time points after the administration. More ideally an App could be developed in which the injection time and the experiences/vital signs of the patient have to be filled in at certain time slots. This should be done by the patients or family. An internal alarm system should identify the problems and alert the HCPs. Alternatively, administration service could be combined and SC trastuzumab could be given at the practice of the GP by either the nurse or the GP. At the moment, there is insufficient remuneration for a nurse in the home setting. The increase of remuneration will be an additional health cost for the government. Another major hurdle is the transport of the drug from hospital pharmacy to the patients’ home. The preparation is done in the hospital pharmacy. Once the syringe is prepared it is stable for 48 hours at a temperature of 2°C – 8°C. If the product is exposed in diffused daylight it remains stable for only 6 hours at ambient temperature (max. 30°C). A solution could be that the patient buys the SC formulation in the public pharmacy and that the nurse prepares the SC trastuzumab at home. Administering a medication at home with possible adverse events, requires that the responsible caregivers of the local network (GPs and nurses) are informed. Lastly, there is additional work for the organisation of checking the medical file, requesting additional investigations, prescribing the medication and sending it to the pharmacy.

The organisation of SC trastuzumab in a home setting requires a good cooperation with the HCPs involved. The introduction of SC at home is feasible and highly appreciated by the patients. A business plan is needed for the administration at home. All the different administrative and organisation steps should have a remuneration. At a first glance, it appears also financially interesting. However, when you calculate all the steps of the stakeholders it is dubious if this is true. The final option would be to teach the patient how she could inject herself and to educate her about the risks. In July 2015, there was a project call from the government with a focus on re-organization of hospital financing and hospital landscape and in May 2016 the project of hospitalization at home was selected. The project “trastuzumab SC at home” has been submitted by several hospitals. This pilot project will further assess the possibilities of SC trastuzumab administration at home.

In Belgium, there is the option of intravenous AclastaR administration at home by the Remecare Online Health Management (Remecare, 2016). Any treating physician can apply for these professional services by which a nurse administrates Aclasta R IV at home. Register at the secured platform of Remecare activates a treatment reminder for both the patient and the physician. This reminder service is provided by the Novartis, this is the company who sells AclastaR (Remecare, 2016). Alternatively if a treating physician wishes to treat a patient with AclastaR by the local nurse or at day care unit the use of the reminder service of Novartis is also available after registration at www.myactive.be.

A new proposal which will be in practice soon is the assessment and blood sampling at home a few days before the planned chemotherapy session by the RemeCoach application from Remedus (Remedus, 2016; RemeCare, 2016). The blood results and the assessment will become available in the digital medical hospital file. The treating physician will prescribe the chemotherapy or targeted therapy based on the results and the following day the patient receives the medication in a day care unit. Due to the RemeCoach there is a follow-up of all important parameters and side effects, which allows a quick intervention when needed (Remedus, 2016). The extra service requires an additional, not reimbursed, cost for the patients of an estimated amount of €50. Recently the evaluation of an interactive electronic patient-reported outcome (e-PRO) system in outpatient setting with oral chemotherapy was presented (Rasschaert et al., 2016). An e-PRO system collects the patient-reported outcome (PROMS) by electronic applications (computers, smartphones or telephone systems). The e-PRO runs on a computer or smartphone and represents a sort of electronic diaries with the possibility to answer a question with a set of predefined options. The telephone e-PRO is the same, but then based on an interactive voice system. Instead of accessing the computer/ smartphone the patient calls a dedicated number. The acceptability appears to be high, even in elderly patients and in patients not familiar with computers or smartphones. The e-PRO for monitoring patients receiving chemotherapy is interesting. It allows to collect symptoms or particular adverse events, which require medical intervention.

Another point of discussion is the financial impact for the hospital, because the treatment is no longer administered in the oncological day care clinic. In the presented studies, this issue was not taken into consideration, because there are differences from country to country in terms of reimbursement. This aspect reflects a potential reduction of income for the hospital and/or treating physician. The government on the other hand may perceive this as a reduction in unnecessary costs.

## Conclusive remarks

From a scientific point of view both formulations of trastuzumab are equally safe and effective. The administration of SC perceptive at home is also feasible. The patients and HCPs prefer SC trastuzumab administration above IV. Administrating trastuzumab SC in a hospital setting instead of IV will reduce the patient chair time and the health care professional time. Both will increase the capacity of an oncological day clinic and reduce the waiting list. The shift from IV to SC also reduces waste and consumables. When trastuzumab is administered at home there is further increase in patient’s satisfaction. For the future, it is likely that the patient will inject herself SC under the guidance of a “you tube” film and e-PRO. In general the assessment at home for chemotherapy will improve the patient’s care. The key question is who will pay for the organisation at home or the shifting in care from a hospital to a home setting. Professional organisations like Remedus fill in the “home” organisation and are paid by the patient. The organisation of for instance checking the e-PRO, the medical file, prescribing the mediation, transporting the medication, informing the HCPs and acting on adverse events at home still costs time and money. Who will pay for all these steps? The system will only change if there is an organisation, which has a high satisfaction rate for all the stakeholders. The organization of the home administration, the teaching of the patients and the HCPs will have a cost price and it will be interesting to see how the hospital and government will deal with this situation in the time of budget costs. It will be an exercise of finding the balance between maintaining or improving the quality of care without increasing additional treatments or administrative actions
